# Determinants of patient satisfaction 5 years after dual-mobility trapeziometacarpal joint implant arthroplasty

**DOI:** 10.1177/17531934261468204

**Published:** 2026-07-24

**Authors:** Daniel B Herren, Kei H Mathis, Stephan Schindele, Miriam Marks

**Affiliations:** 1 Department of Hand Surgery, Schulthess Klinik, Zurich, Switzerland; 2 Department of Teaching, Research and Development, Schulthess Klinik, Zurich, Switzerland

**Keywords:** Hand surgery, patient-reported outcomes, thumb carpometacarpal joint, thumb carpometacarpal osteoarthritis, touch dual-mobility prosthesis

## Abstract

**Introduction:**

The primary aim of this registry-based case series was to identify determinants of patient satisfaction 5 years after dual-mobility trapeziometacarpal joint (TMJ) implant arthroplasty. Secondary aims included the assessment of 5 year outcomes and implant survival.

**Methods:**

Patients who underwent primary implantation of a dual-mobility TMJ prosthesis were included. Satisfaction at 5 years was assessed using a five-point Likert scale and analysed using a top-box approach. Determinants of being ‘very satisfied’ were evaluated using univariate logistic regression. Pain (numeric rating scale), hand function (brief Michigan Hand Outcomes Questionnaire, MHQ) and key pinch strength were assessed. Changes in outcomes between baseline and 5 years were analysed using a linear mixed-effects model. Implant survival was estimated using the Kaplan–Meier method.

**Results:**

Of the 125 patients who underwent surgery, 108 were available for the 5 year analysis and 94 completed the satisfaction questionnaire. Of the latter, 87 (93%) were ‘satisfied’ or ‘very satisfied’ with the treatment outcome. Lower pain levels and higher brief MHQ scores at 5 years were significantly associated with being ‘very satisfied’. Baseline characteristics, complications and revision surgery were not associated with satisfaction. Baseline pain, brief MHQ and key pinch strength improved significantly by 5 years. Five revision surgeries occurred, resulting in an estimated 5 year implant survival of 96% (95% CI 92 to100).

**Conclusion:**

Our results support existing evidence that dual-mobility TMJ arthroplasty yields high patient satisfaction, significant clinical improvement and excellent implant survival at 5 years. Pain relief and improved hand function appear to be key determinants of patient satisfaction.

**Level of Evidence:**

IV

## Introduction

The introduction of dual-mobility trapeziometacarpal joint (TMJ) joint prostheses has greatly influenced clinical practice in hand surgery in recent years (Herren et al., 2024). Medium-term results indicate that patients with TMJ osteoarthritis (OA) generally report high levels of satisfaction with this procedure. At 3–8 years of follow-up, 95–100% of patients reported being satisfied or very satisfied with their treatment outcome (Falaise and Boulat, 2025; Lussiez et al., 2021). In a separate cohort, the median satisfaction score at 5 years after single-mobility implant arthroplasty was 9 out of 10 points (Bonhof-Jansen et al., 2026).

Dual-mobility implant survival also appears promising, although long-term data are still lacking. Reported survival rates lie between 95 and 98% at follow-up periods ranging from 3 to 6.5 years (Dass et al., 2026; Falaise and Boulat, 2025; Frittella et al., 2025; Lussiez et al., 2021; Tchurukdichian et al., 2021). Despite these favourable outcomes, a subset of patients continues to report unsatisfactory results. Evidence from small studies indicates that patient satisfaction is influenced by factors such as pain, strength, hand appearance, recovery time and postoperative complications (Dietrich et al., 2025; Nietlispach et al., 2025). However, a comprehensive analysis of the determinants of both satisfaction and dissatisfaction remains absent. A better understanding of these factors may help surgeons refine patient selection and improve preoperative counselling.

The primary aim of our registry-based case series study was to identify determinants of patient satisfaction 5 years after dual-mobility TMJ implant arthroplasty. Secondary aims were to evaluate clinical and patient-reported outcomes as well as 5 year implant survival.

## Methods

### Patients and setting

This study represents a follow-up to our previous analysis of 2 year outcomes, for which full methodological details are available (Herren et al., 2023). For the current analysis, we included all patients from our single-centre registry (Mathis et al., 2025) who underwent primary implantation of the Touch^®^ prosthesis (Keri Medical, Plan-les-Ouates, Switzerland) between June 2018 and December 2020 and who had completed the 5 year follow-up. Patients who received the implant as a revision procedure or who did not provide written informed consent were excluded.

The data analysis was approved by the local ethics committee and conducted according to the RECORD (REporting of studies Conducted using Observational Routinely-collected health Data) statement (Benchimol et al., 2015).

### Intervention

The prosthesis was implanted using the standard dorsolateral approach technique (Lussiez et al., 2021). A spheric cup was used for all patients. At the discretion of each surgeon, the capsule was either resected or sutured. After 3–5 days of postoperative immobilization, patients began with active non-loaded mobilization. Patients were allowed to return to unrestricted daily activities after the 6 week radiographic control.

### Outcome measures

All patients were assessed before surgery (i.e. baseline) and at 6 weeks, 3 months and 1, 2 and 5 years postoperatively. At each follow-up, patients completed standardized questionnaires and underwent clinical assessment by the surgeon. A study assistant contacted patients who had not attended the follow-up to ask whether the implant was still *in situ*.

The primary outcome measure was patient satisfaction with the treatment result at 5 years. Patients rated their satisfaction on a five-point Likert scale, with response options ranging from *very satisfied*, *satisfied*, *neither satisfied nor dissatisfied*, *dissatisfied* to *very dissatisfied*. A similar satisfaction measure with comparable response options has demonstrated high reliability and construct validity (De Ridder et al., 2021).

Patients rated their pain at rest and during activities on a numeric rating scale ranging from 0 to 10, where 0 indicates no pain and 10 maximum pain. Patient-reported hand function was quantified with the brief Michigan Hand Outcomes Questionnaire (MHQ) (Knobloch et al., 2012; Waljee et al., 2011). The score ranges from 0 to 100 with higher scores indicating better hand function. The EuroQol-5 Dimensions-5 Level (EQ-5D-5L) questionnaire was used to measure overall quality of life and the anxiety/depression dimension of the EQ-5D-5L (item 5) was used as a proxy measure for mental health (Herdman et al., 2011). One measurement of maximum key pinch strength was done in a standardized sitting position using a pinch gauge (B&L Engineering, Santa Ana, USA). Flexion and extension of the metacarpophalangeal joint was measured with a goniometer. Standard posteroanterior and lateral radiographs of the TMJ joint at follow-up were evaluated for implant loosening, migration, and/or dislocation.

Complications, subsequent soft tissue surgeries and implant revisions were recorded for each patient.

### Statistics

For descriptive statistics, means, standard deviations and 95% CIs were calculated. Because satisfaction responses typically exhibit a pronounced ceiling effect (Wilson et al., 2023), we applied a top-box approach (Brown, 2020; North and Tulledge-Scheitel, 2019). This approach is a simple and widely used method for analysing patient-reported satisfaction when responses are highly skewed (i.e. where a high proportion of participants choose the most positive option). Thus, we defined the ‘top-box’ as the proportion of patients who indicated being *very satisfied*, and compared this group to the proportion of patients who indicated being *satisfied* or worse. Univariate logistic regression was used to identify determinants of being *very satisfied*. Independent variables were selected based on clinical relevance and included both baseline and 5 year data, unless otherwise stated: age, sex, Eaton stage of OA, dominant hand affected, employment status, surgeon experience according to Tang and Giddins (2016), prior surgery on the same hand, occurrence of complications, pain, brief MHQ, quality of life, mental health, key pinch, metacarpophalangeal extension and implant loosening or migration (the latter was assessed only at the 5 year follow-up). Odds ratios were calculated. Characteristics and outcomes of patients who were not *very satisfied* were compared with those of the top-box group using the Mann–Whitney *U*-test. Characteristics of patients who were *dissatisfied* and *very dissatisfied* patients were also examined descriptively in greater detail. Changes in clinical and patient-reported outcomes between baseline and 5 years were analysed using a linear mixed-effects model. Missing values were not imputed; however, we performed a non-responder analysis. We compared the outcomes of patients who did not complete the 5 year satisfaction question with those who did using the Mann–Whitney *U*-test for interval-scaled and ordinal outcomes, and Fisher’s exact test for binary outcomes.

Five-year implant survival was estimated using the Kaplan–Meier method. Patients were censored at the time of revision or at the time of dropout from the registry for other reasons such as participation withdrawal.

## Results

During the study period, 125 patients were documented in our local registry who underwent a TMJ implant arthroplasty and provided written informed consent. For the 5 year analysis, 108 patients were available (86%) (Figure 1). To our knowledge, none of the patients with missing follow-up had undergone revision surgery elsewhere. At 5 years, 94 patients completed the satisfaction questionnaire; 93% were *satisfied* or *very satisfied* with the treatment result (Table 1). The non-responder analysis revealed that patients with missing 5 year data were 7 years older than patients who completed the questionnaire (*p* ⩽ 0.05). There were no differences in other baseline characteristics or 1 year outcomes between responders and non-responders (Table 2). The mean patient age at surgery for those finally analysed was 63 years (SD 7.8) and 68% were female (Table 3).

**Figure 1.**
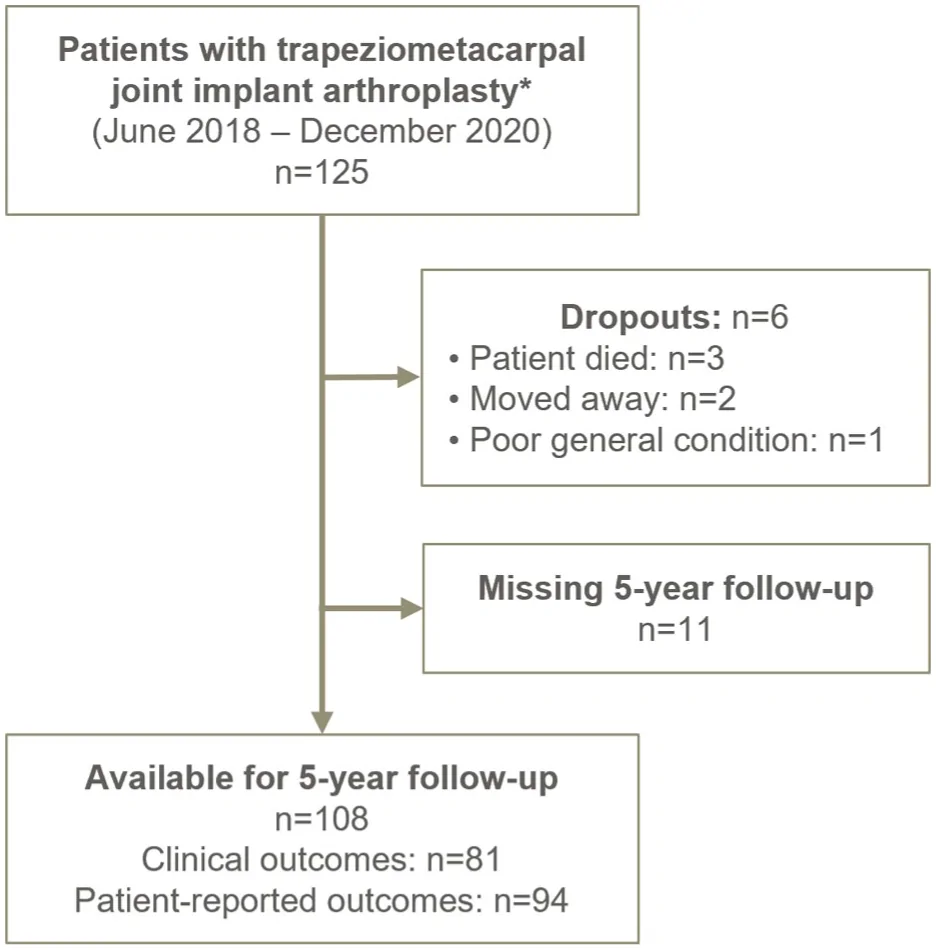
Patient recruitment diagram. *Patients who provided written informed consent for the use of their data.

**Table 1.** Satisfaction with the treatment outcome at 5 years of 94 patients who completed the satisfaction question.

Response	*N* (%)
Very satisfied (= top-box)	75 (80)
Satisfied	12 (13)
Neither satisfied nor dissatisfied	4 (4)
Dissatisfied	3 (3)
Very dissatisfied	0 (0)

**Table 2.** Non-responder analysis comparing baseline characteristics and 1 year outcomes of 94 patients who completed the 5 year satisfaction question with those who did not (*n* = 31). Median (interquartile ranges) are shown if not indicated otherwise.

Variable	Responder		Non-responder		*p*-Value
	*n*	Value	*n*	Value	
Age at surgery (years)	94	61 (9)	31	68 (12)	**0.04**
Sex, female [*n* (%)]	94	65 (69)	31	20 (65)	0.66
Previous surgeries [*n* (%)]	89	10 (11)	31	1 (3)	0.29
Pain during activities baseline	88	8.0 (1.0)	25	8.0 (2.0)	0.47
Pain during activities at 1 year	88	1.0 (2.0)	19	1.0 (2.5)	0.54
Brief MHQ baseline	88	45 (19)	25	44 (21)	1.0
Brief MHQ at 1 year	88	92 (19)	19	94 (18)	0.84
Key pinch baseline (kg)	92	4.3 (3.1)	31	4.0 (3.3)	0.44
Key pinch at 1 year (kg)	79	7.0 (2.0)	24	6.3 (3.0)	0.37
*Satisfied* or *very satisfied* at 1 year [*n* (%)]	88	82 (93)	19	18 (95)	0.55

MHQ, Michigan Hand Outcomes Questionnaire.

Bold values indicate significant *p*-values.

**Table 3.** Comparison of baseline and 5 year clinical and patient-reported outcomes between *very satisfied* vs. not *very satisfied* patients. Means and 95% CIs are shown, unless otherwise indicated.

Variable	*Very satisfied* (*n* = 75)	Not *very satisfied* (*n* = 19)	*p*-Value
Age at surgery (years)	63 (61 to 65)	61 (57 to 64)	0.21
Sex, female (%)	71	63	0.53
Previous surgeries (%)	12	5	0.36
Pain at rest baseline	5.3 (4.8 to 5.9)	4.5 (3.5 to 5.5)	0.21
Pain at rest at 5 years	0.3 (0.1 to 0.4)	1.8 (1.1 to 2.6)	**⩽ 0.001**
Pain during activities baseline	7.6 (7.1 to 8.0)	7.3 (6.7 to 7.9)	0.34
Pain during activities at 5 years	0.4 (0.3 to 0.6)	3.6 (2.6 to 4.7)	**⩽ 0.001**
Brief MHQ baseline	44 (40 to 47)	48 (43 to 53)	0.19
Brief MHQ at 5 years	92 (89 to 95)	71 (64 to 78)	**⩽ 0.001**
EQ-5D-5L baseline	0.68 (0.63 to 0.73)	0.73 (0.65 to 0.81)	0.75
EQ-5D-5L at 5 years	0.89 (0.85 to 0.94)	0.80 (0.69 to 0.91)	**⩽ 0.001**
Key pinch baseline (kg)	4.9 (4.3 to 5.6)	5.6 (4.3 to 6.9)	0.36
Key pinch at 5 years (kg)	6.8 (6.4 to 7.3)	7.4 (5.9 to 8.9)	0.66

MHQ, Michigan Hand Outcomes Questionnaire; EQ-5D-5L, EuroQol-5 Dimensions-5 Level questionnaire.

Bold values indicate significant *p*-values.

Patients with lower pain and higher brief MHQ scores at 5 years had a significantly higher odds ratio of being *very satisfied* with the treatment outcome (Figure 2). Neither the baseline variables nor the occurrence of complications or revision surgery showed a significant association with treatment satisfaction at 5 years. Pain, brief MHQ and EQ-5D-5L scores at 5 years were significantly improved for *very satisfied* patients than for those who were not *very satisfied* (*p* ⩽ 0.01) (Table 3). The three dissatisfied patients all had high pain (numeric rating scale ⩾ 5) that limited their daily activities.

**Figure 2.**
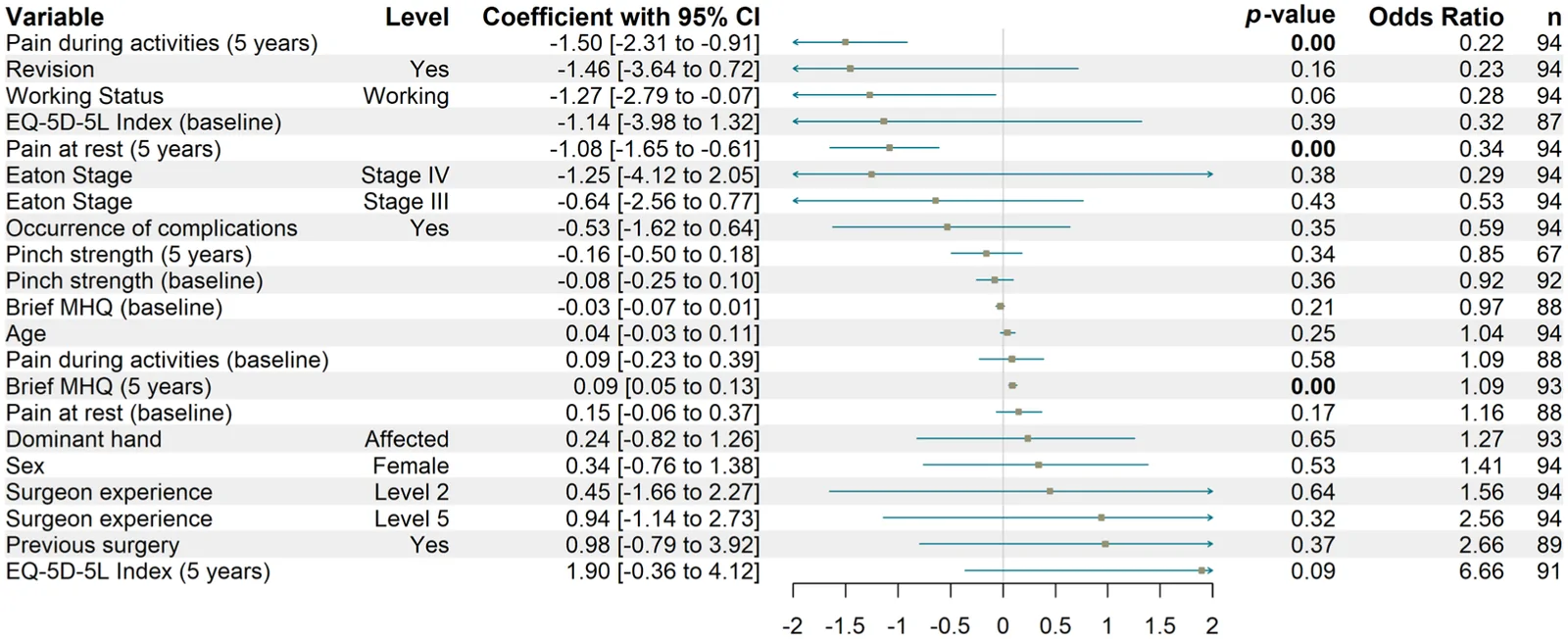
Forest plot showing univariate logistic regression analysis with coefficients (square points) and their 95% CIs (horizontal lines) and odds ratios of different factors for being *very satisfied* with the treatment outcome at 5 years (*n* = 75). Significant *p*-values are shown in bold.

Pain at rest, pain during daily activities, the brief MHQ score and key pinch strength improved significantly from baseline to 5 years (*p* ⩽ 0.001) (Figure 3).

**Figure 3.**
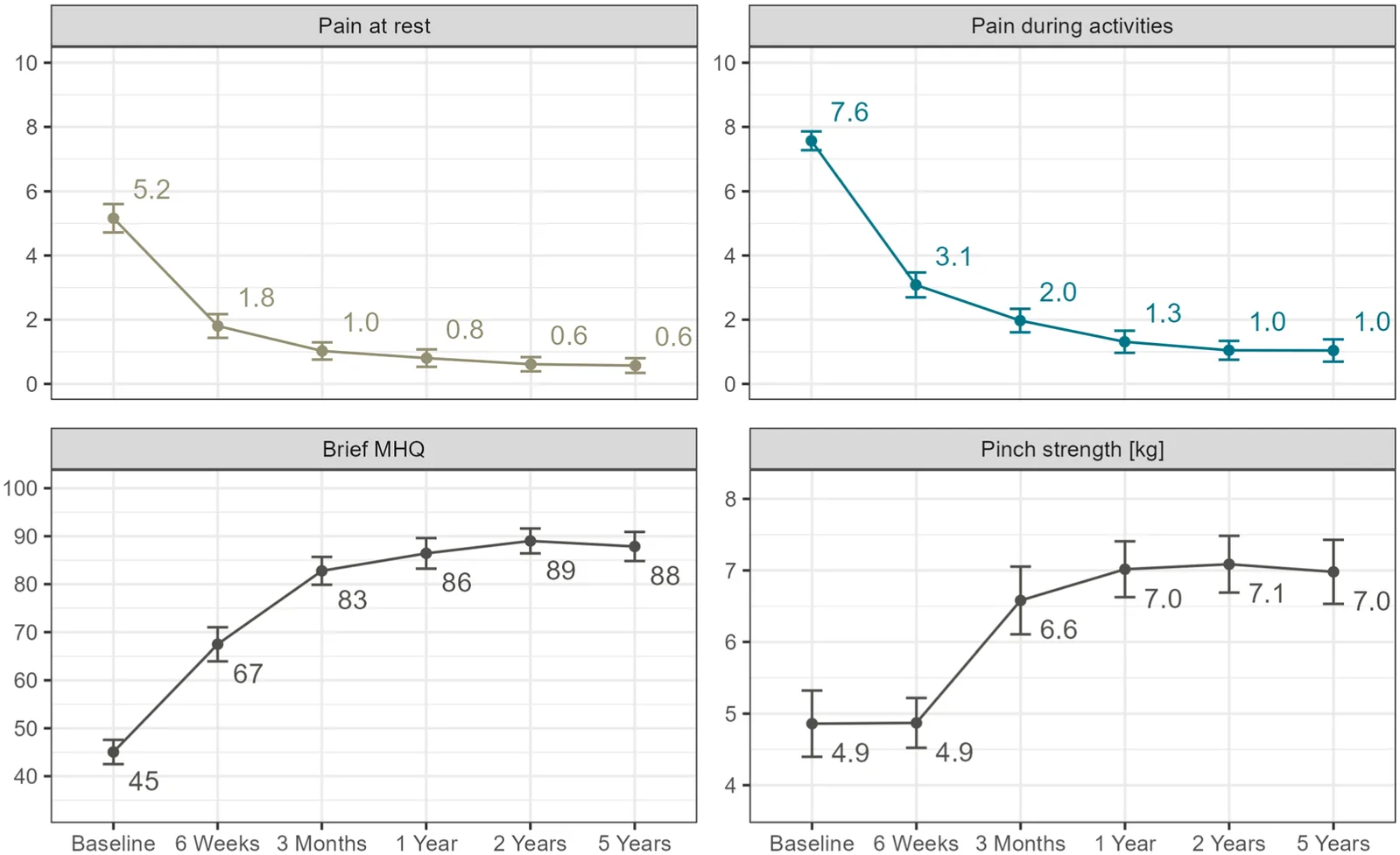
Mean values (points) and 95% CIs (whiskers) for pain at rest, pain during activities, the brief Michigan Hand Outcomes Questionnaire (MHQ) and key pinch strength throughout the 5 year postoperative period.

Five patients (four female, one male) had to undergo revision surgery at a mean time of 17 months (range 0.4–28) after the primary intervention. The details of 4 patients who underwent revision up to 2 years for cup loosening (*n* = 2), dislocation (*n* = 1) and painful impingement (*n* = 1) have been previously described (Herren et al., 2023). Two were converted to a resection arthroplasty and in two cases, changing components resulted in the prostheses being retained. The fifth patient fell on her hand, which resulted in cup loosening and required conversion to resection arthroplasty 28 months after the initial surgery. Five years after revision surgery, two patients were *very satisfied*, one was *satisfied* and one was *neither satisfied nor dissatisfied* with the treatment outcome; the fifth patient had missing data. The estimated 5-year survival rate was 96% (CI 92 to 100%) (Figure 4).

**Figure 4.**
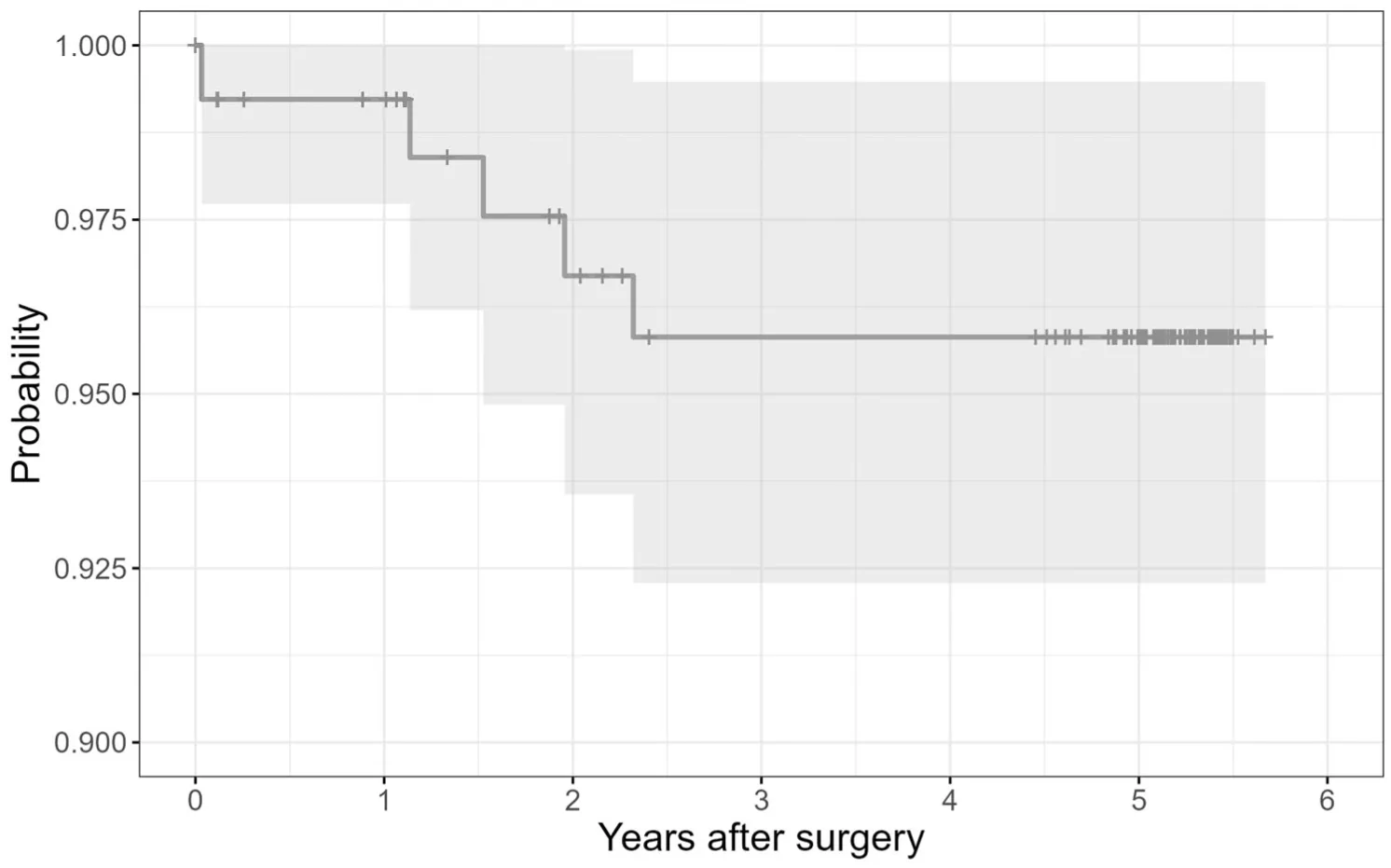
Kaplan–Meier plot showing the estimated 5 year implant survival rate of 96%. The grey area represents the 95% CI (92 to 100%). Vertical marks indicate censored patients, namely those who dropped out owing to reasons other than revision.

In addition to the 17 complications reported in the 2 year paper – most commonly de Quervain syndrome (*n* = 6) and trigger finger (*n* = 5) – one further case of de Quervain syndrome occurred 2.1 years postoperatively after a period of intensive music making. At the 5 year follow-up, only one additional radiolucent line was noted in a patient who sustained a distal radius fracture, but this finding was not accompanied by any thumb symptoms, and the patient was very satisfied with the treatment outcome (Figure 5).

**Figure 5.**
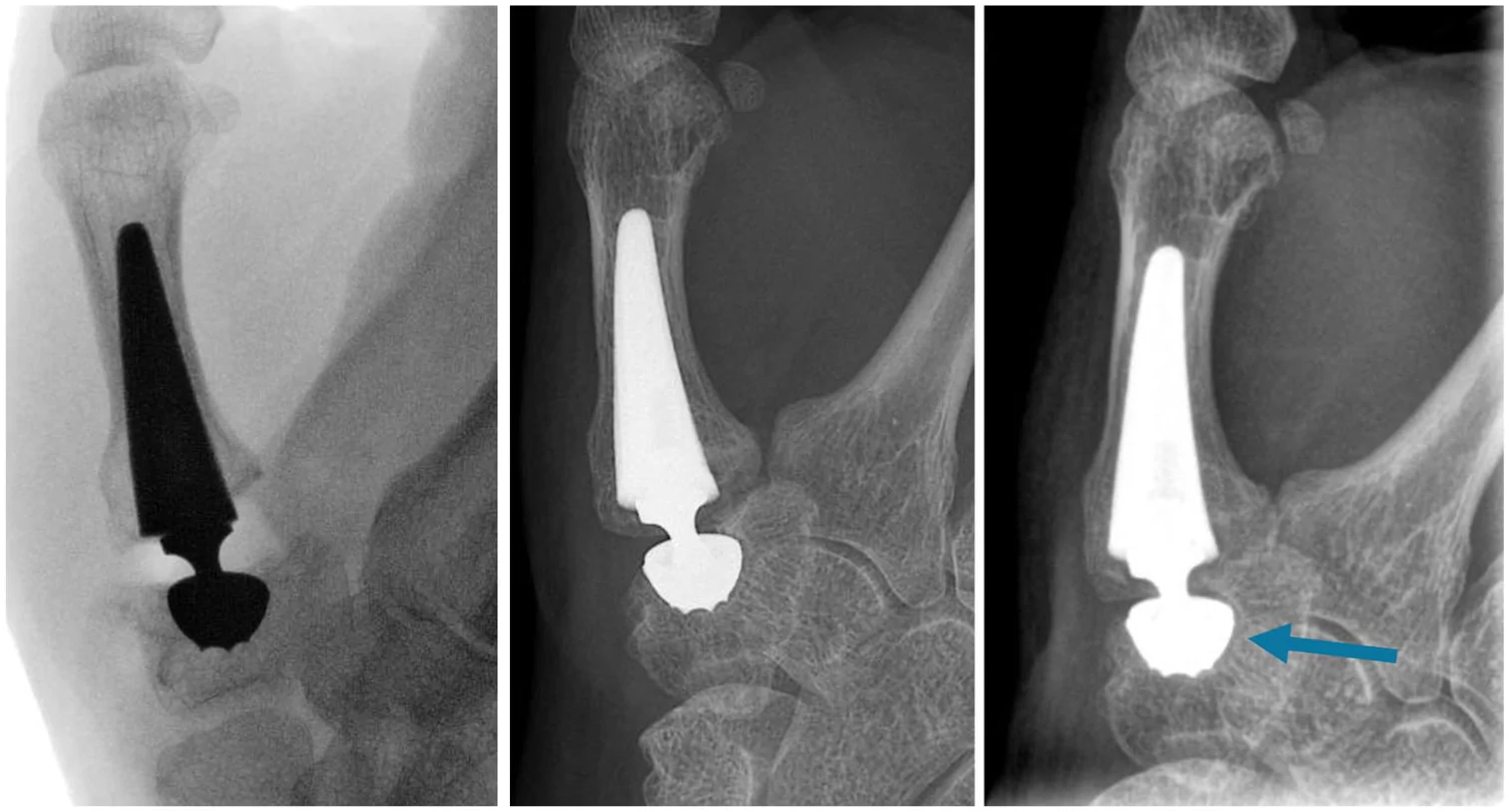
Left: an intraoperative image showing a 59-year-old male undergoing trapeziometacarpal joint dual-mobility implant arthroplasty. Middle: 2 years post-surgery, the implant is stable. The patient sustained a distal radius fracture 2.4 years after the initial surgery. Right: 5 years after the initial surgery, a radiolucent line (arrow) is visible on the radiograph; however, the patient is pain-free and very satisfied with the outcome of the arthroplasty procedure.

## Discussion

The results of this study show that 93% of patients were *satisfied* or *very satisfied* with the treatment outcome 5 years after TMJ dual-mobility implant arthroplasty. Lower pain levels and better hand function at follow-up were associated with higher patient satisfaction, whereas neither baseline variables nor the occurrence of complications were significantly associated with satisfaction at the 5 year mark. Both clinical and patient-reported outcomes improved significantly compared to baseline, and implant survival was high with a 5 year survival rate of 96%.

The high satisfaction observed in our study aligns with previous reports on dual-mobility TMJ prostheses, in which 95–100% of patients were satisfied or very satisfied 3–8 years postoperatively (Falaise and Boulat, 2025; Lussiez et al., 2021). Implant arthroplasty also appears to yield higher satisfaction than resection arthroplasty. Among patients who had undergone implant arthroplasty on one hand and resection arthroplasty on the other, satisfaction was higher for implant (86%) than for resection arthroplasty (57%) (Nietlispach et al., 2025). Similarly, a randomized trial demonstrated higher satisfaction after implant arthroplasty compared with trapeziectomy at 5-year follow-up (Bonhof-Jansen et al., 2026).

Lower pain levels and better hand function at follow-up were strongly associated with higher patient satisfaction, consistent with findings in other orthopaedic hand disorders (Choi et al., 2022; De Ridder et al., 2022; Rogers et al., 2023). The results suggest that pain relief is a key factor in patient-perceived success following surgical treatment for TMJ OA. In our cohort, the few patients who were dissatisfied reported persistent pain that interfered with daily activities, further highlighting the central importance of effective pain control. However, we did not identify any baseline variables such as age, preoperative pain or hand function that predicted satisfaction. Previous studies have suggested that higher baseline brief MHQ scores, positive coping strategies and high preoperative expectations may contribute to better satisfaction outcomes (Arends et al., 2026; Choi et al., 2022; De Ridder et al., 2022; Lynch et al., 2024). We think that the relatively homogeneous baseline characteristics in our cohort and the pronounced ceiling effect in satisfaction ratings may have limited the ability to detect such associations. Additionally, complications have been reported as a determinant of satisfaction in earlier work (Arends et al., 2026), but this was not the case in our study. This discrepancy may be explained by the nature of the complications observed in our cohort, which were primarily soft tissue-related and successfully managed with steroid injections or minor soft tissue procedures (Herren et al., 2023).

The high survival rate observed in our study is consistent with previous reports showing survival rates between 95 and 98% at follow-up periods of 3–6.5 years (Dass et al., 2026; Falaise and Boulat, 2025; Frittella et al., 2025; Lussiez et al., 2021; Tchurukdichian et al., 2021). These findings further strengthen the growing body of evidence that dual-mobility TMJ implant arthroplasty is a safe medium-term treatment option. Notably, the risk of revision does not appear to exceed that of resection arthroplasty, for which medium-term revision rates of 2–5% have been reported (Latelise et al., 2024; Mattila and Waris, 2019). Most revisions in this cohort occurred within the first 2 years and were related to cup loosening, dislocation or impingement. Three revisions were probably related to suboptimal surgical technique, highlighting the considerable learning curve involved in prosthesis implantation (Herren et al., 2025). Another complication that may arise more than 2 years postoperatively is polyethylene liner fracture, particularly in active males with high key pinch strength. Early detection is essential, as timely head and liner exchange may allow the implant to be preserved, whereas delayed treatment can lead to metallosis, cup loosening and ultimately implant removal (Duerinckx et al., 2026).

### Limitations

First, the analysis of registry data used for routine clinical documentation results in missing values and incomplete and missing follow-up data. This introduces the possibility of selection bias. As we have contacted all patients who did not attend their follow-up appointments and asked about the condition of their implant, we assume that we have not missed any revisions. However, we cannot discount the possibility that patients may have undergone revision surgery elsewhere. Second, the implant arthroplasties were performed by seven different surgeons, and small variations in surgical technique may have influenced the outcomes. Furthermore, the limited variability in baseline characteristics, combined with a marked ceiling effect in satisfaction scores, may have reduced the sensitivity to identify associations with baseline factors. Finally, satisfaction ratings were converted from an ordinal to a binary outcome, which may have led to some loss of information. However, because only a few patients reported less than very high satisfaction, an ordinal analysis was not feasible, and multivariable modelling was avoided to reduce the risk of overfitting.

In conclusion, our results align with existing evidence indicating that dual-mobility implant arthroplasty yields high patient satisfaction, excellent clinical outcomes and high implant survival at 5 years. This supports its use as a safe and effective treatment for patients with TMJ OA. Our findings highlight the importance of achieving adequate pain relief and functional improvement to optimize patient satisfaction after TMJ arthroplasty.
